# Plasma amyloid-β oligomerization assay as a pre-screening test for amyloid status

**DOI:** 10.1186/s13195-021-00873-w

**Published:** 2021-07-26

**Authors:** Rosha Babapour Mofrad, Philip Scheltens, SangYun Kim, Sungmin Kang, Young Chul Youn, Seong Soo A. An, Jori Tomassen, Bart N. M. van Berckel, Pieter Jelle Visser, Wiesje M. van der Flier, Charlotte E. Teunissen

**Affiliations:** 1 Neurochemistry Laboratory and Biobank, Department of Clinical Chemistry, Amsterdam Neuroscience, VU University Medical Center, Amsterdam UMC, Amsterdam, The Netherlands; 2 Alzheimer Center & Department of Neurology Amsterdam, Neuroscience Campus Amsterdam Neuroscience, VU University Medical Center, Amsterdam UMC, Vrije Universiteit Amsterdam, Amsterdam, the Netherlands; 3 Department of Neurology, Seoul National University Bundang Hospital, Seoul National University College of Medicine, Gyeonggi-do, Republic of Korea; 4 Department of Research and Development, PeopleBio Inc, Seongnam-si, Republic of Korea; 5 Department of Neurology, Chung-Ang University College of Medicine, Seoul, Republic of Korea; 6 Department of Bionanotechnology, Gachon University, Incheon, Republic of Korea; 7 Department of Radiology & Nuclear Medicine, Amsterdam Neuroscience, Vrije Universiteit Amsterdam, Amsterdam UMC, Amsterdam, The Netherlands; 8 Department of Psychiatry & Neuropsychology, School for Mental Health and Neuroscience, Maastricht University, Maastricht, The Netherlands; 9 Department of Epidemiology and Biostatistics, Amsterdam UMC, VU University Medical Center, Vrije Universiteit Amsterdam, Amsterdam, The Netherlands

**Keywords:** Blood-based biomarker, Plasma Aβ oligomer, Amyloid status, Multimer detection system, Long-term storage

## Abstract

**Objective:**

We assessed the performance of plasma amyloid oligomerization tendency (OAβ) as a marker for abnormal amyloid status. Additionally, we examined long-term storage effects on plasma OAβ.

**Methods:**

We included 399 subjects regardless of clinical diagnosis from the Amsterdam Dementia Cohort and European Medical Information Framework for AD project (age, 63.8 ± 6.6; 44% female). Amyloid status was determined by visual read on positron emission tomography (PET; n_abnormal_ = 206). Plasma OAβ was measured using the multimer detection system (MDS). Long-term storage effects on MDS-OAβ were assessed using general linear models. Associations between plasma MDS-OAβ and Aβ-PET status were assessed using logistic regression and receiver operating characteristics analyses. Correlations between plasma MDS-OAβ and CSF biomarker levels were evaluated using Pearson correlation analyses.

**Results:**

MDS-OAβ was higher in individuals with abnormal amyloid, and it identified abnormal Aβ-PET with an area under the curve (AUC) of 0.74 (95% CI, 0.67–0.81), especially in samples with a storage duration < 4 years. Combining APOEe4 and age with plasma MDS-OAβ revealed an AUC of 81% for abnormal amyloid PET status (95% CI, 74–87%). Plasma MDS-OAβ correlated negatively with MMSE (r = − 0.29, p < .01) and CSF Aβ42 (r = − 0.20, p < 0.05) and positively with CSF Tau (r = 0.20, p = 0.01).

**Conclusions:**

Plasma MDS-OAβ combined with APOEe4 and age accurately identifies brain amyloidosis in a large Aβ-confirmed population. Using plasma MDS-OAβ as a screener reduced the costs and number of PET scans needed to screen for amyloidosis, which is relevant for clinical trials. Additionally, plasma MDS-OAβ levels appeared affected by long-term storage duration, which could be of interest for others measuring plasma Aβ biomarkers.

## Background

Accumulating evidence shows that small soluble Amyloid-β oligomers (AβOs) are the most toxic and pathogenic form of Aβ species in Alzheimer’s disease (AD) [1, 2]. Many toxicities have been ascribed to AβOs including synaptic dysfunction, induction of tau pathology, neuroinflammation, impaired axonal transport, and neuronal death [3]. In addition, AβOs have shown a better correlation with the presence and degree of cognitive symptoms than Aβ plaque counts [4], suggesting that AβOs might provide a more accurate reflection of clinical presentation than Aβ plaque load.

Currently, proxies of Aβ plaques are measured with high sensitivity and specificity with positron emission tomography (PET) imaging or measurement of cerebrospinal fluid (CSF) Aβ42 concentrations. However, these methods often come with high costs or burden for the patient. Therefore, blood-based biomarkers are considered low-cost and minimally invasive alternatives.

Plasma AβO concentrations or misfolded Aβ oligomeric assemblies have previously shown good diagnostic accuracies in identifying AD from controls (area under the curve (AUC), 0.71–0.80) [5, 6]. Using the multimer detection system (MDS) to measure plasma AβO levels has resulted in even higher diagnostic accuracies (AUC, 0.85–0.87) in discriminating AD dementia patients from controls [7]. However, the ability of plasma amyloid oligomerization tendency measured by the multimer detection platform (MDS-OAβ) to identify individuals with abnormal amyloid status has not yet been studied. This is relevant, because the definition of AD in vivo is shifting to a biological construct and increasingly based on amyloid status [8]. Therefore, we aimed to assess the performance of plasma MDS-OAβ as a marker for abnormal amyloid status.

## Methods

### Subjects

We included 399 subjects from the Amsterdam Dementia Cohort (ADC) and the European Information Framework for AD (EMIF-AD) Preclinical AD project, regardless of clinical diagnosis. Inclusion criteria were met when amyloid PET results were available and the time between plasma sampling and PET scan did not exceed 1 year. During their visit, all subjects underwent comprehensive dementia screening including neurologic examination, laboratory tests, magnetic resonance imaging (MRI), and electroencephalography (EEG) [9, 10]. Clinical diagnosis was established by consensus according to international consensus criteria [8, 11–14], and included mild cognitive impairment (MCI; n = 42), AD dementia (n = 164), non-AD dementia (n = 58), and other disorders (n = 61) including neuropsychiatric disorders, neurological disorders, or individuals with postponed diagnosis. Controls consisted of participants with subjective cognitive decline (SCD; n = 14) and normal controls (NC; n = 60). Normal controls in this study were included from the preclinical AD study [15]. No known familial AD patients were included. CSF and PET results (below) were used to support the AD dementia diagnosis and to define the number of amyloid-positive subjects within each clinical diagnostic group.

### Amyloid status

Amyloid PET status was available in all subjects (n = 399). [18F]Florbetaben (n = 138), [18F]florbetapir (n = 1), [18F]flutemetamol (n = 138), or [11C]Pittsburgh compound B (PiB; n = 122) were used as radioactive amyloid tracers. A Medrad (Warrendale, PA) infusion system was used for tracer infusion. [18F]Florbetapir and [11C]PIB scans were acquired through 90-min dynamic scanning using a PET/CT Ingenuity TF or Gemini TF [Philips Medical Systems, Best, the Netherlands] ([18F]Florbetapir), and ECAT EXACT HR + scanner [Siemens/CTI, Knoxville, TN] ([11C]PIB). Scanning started simultaneously with tracer infusion at approximately 370 MBq [18F]florbetapir and 351 MBq [11C]PiB. [18F]Florbetaben and [18F]flutemetamol scans were acquired through 20-min static PET scanning using a PET/MR and Gemini TF-64 PET/CT scanner, Philips Medical Systems, respectively. Scanning started 90 min after tracer injection at approximately 250 MBq [18F]florbetaben and 180 MBq [18F]flutemetamol. Amyloid status was defined as either abnormal or normal after visual assessment by either one (ADC) or three experienced nuclear medicine physicians (preclinical AD project) where majority vote ruled.

### Cerebrospinal fluid analysis

CSF Aβ42 was measured using two analytical methods: Innotest and Euroimmun ELISAs. Innotest ELISAs were used to measure levels of CSF amyloid beta 1-42, total Tau (Tau) and Tau phosphorylated at threonine 181 (pTau) for 268 subjects (Fuijirebio, Ghent, Belgium). CSF Aβ levels were corrected for the drift seen throughout CSF analysis years [16]. Euroimmun beta-amyloid ELISAs (Euroimmun, Lübeck, Germany/ADx Neurosciences, Ghent, Belgium) were used to measure levels of CSF Aβ1-40 and 1-42 of normal controls (n = 60). All CSF samples were measured centrally at the Neurochemistry Laboratory of Amsterdam UMC.

### Apolipoprotein E status

APOE genotyping was performed using LightCycler ApoE mutation Detection Kit (Roche Diagnostics, GmbH, Mannheim, Germany), after isolation of genomic DNA from ethylenediaminetetraacetic acid (EDTA) plasma (Qiagen, Venlo, the Netherlands). APOEe4 status was dichotomized into APOEe4 allele carriers (i.e., at least one APOEe4 allele) and non-carriers (i.e., no APOEe4 allele).

### Plasma sampling and analysis

Blood plasma was collected in an EDTA vacutainer tube through venipuncture using standardized in-house protocols. EDTA plasma was centrifuged at 1800×*g* for 10 min at room temperature and stored in 0.5 ml polypropylene tubes at – 80 °C in the Amsterdam UMC biobank. Prior to analysis, plasma aliquots were thawed at 37 °C for 15 min. Peoplebio Inc. measured MDS-OAβ levels in all plasma samples centrally using the multimer detection system, which is CE marked, approved by the Korean FDA and under commercialization for the Asian and European markets. The assay essentially is an ELISA assay, with the exception that samples and standards were mixed with a proprietary amyloid beta protein mixture before starting the sandwich procedure. Results are expressed as ratio of the concentration calculated in the standard curve for each sample over the average results obtained for two internal standards. All samples were analyzed twice in singlet, on 2 consecutive days. Intra-assay variations were below 10% and 2% of the samples (n = 8) showed interassay variations higher than 20% coefficient of variation (CV) (these samples were included in the analyses). All samples were above the lower limit of quantification (LLOQ; 0.239 ng/ml). Hemolytic samples (n = 15) were excluded, as hemoglobin might interfere with the MDS-OAβ signal [17]. The exact MDS method was described in detail previously [18]. The reproducibility of MDS-OAβ test confirmed by testing the control materials with four concentrations (highly concentrated positive, medium concentrated positive, low concentrated positive and negative) with three different LOTs, two different instruments/site, three different testers for 5 days with five replicate per each sample. As a result, all 225 positive sample test results were positive and all 75 negative sample test results were negative, showing 100% reproducibility, and calculated CV% of MDS ratio for total and between LOT, tester, and instrument (site) was approximately 5%. There was no interference of albumin, hemoglobin, or biliburin, nor cross reactivity of Mutant Aβ1-42 monomer peptide, Aβ4-42 peptide, Aβ9-42 peptide, and Aβ1-24 peptide with a concentration of 1.25 ng/ml.

### Statistical analyses

SPSS version 24 (IBM, Armonk, NY) was used for all statistical analyses, and data were visualized using R version 3.5.2 (“eggshell igloo”). Subject characteristics were compared between amyloid normal and abnormal subjects, using Student’s t-test, chi-squared test, and Mann-Whitney U tests where appropriate. Plasma MDS-OAβ was normalized using a 2-step transformation [19]. Upon visual inspection, we recently observed a possible effect of long-term storage on plasma MDS-OAβ levels (data not published). As plasma samples which are used for research purposes might have a long storage period after biobank retrieval, we additionally assessed the effect of long-term storage on plasma MDS-OAβ levels by using general linear models (GLM) with factors sample storage period, PET amyloid status, and their 2-way interactions. In case of a significant 2-way interaction between storage period in years and PET amyloid status, we performed GLM for plasma MDS-OAβ stratified for storage period based on the median storage period (4 years). Differential expression of plasma MDS-OAβ levels between normal amyloid and abnormal amyloid subjects was evaluated using GLM with plasma MDS-OAβ as outcome measure, amyloid status as factor, and age, sex, APOEe4 status, and cohort as covariates. Syndrome diagnosis was not a confounder and therefore not included as a covariate. Pearson correlation analyses were used to assess correlations between plasma MDS-OAβ and Mini Mental State Examination (MMSE) scores as measure of global cognition, or levels of CSF Aβ42, Tau, and pTau measured by Innotest ELISA. The potential of the plasma Aβ oligomer assay to identify PET abnormal amyloid status was assessed by receiver operating characteristic (ROC) analysis using predicted values of binary logistic regression models. The area under the curve (AUC) and corresponding sensitivities and specificities were calculated at an optimal cut-off for each model using Youden J’s index (sensitivity + specificity − 1). The models contained (1) plasma MDS-OAβ, (2) age and APOEe4 status, or (3) plasma MDS-OAβ combined with age and APOEe4 status. AUC’s between models were compared using DeLong analysis [20]. Additionally, to assess the performance of the plasma MDS-OAβ assay in early AD stages, analyses were repeated in a subgroup of pre-dementia subjects including controls and MCI subjects. Lastly, we investigated how the use of the plasma MDS-OAβ marker can reduce the costs and number of PET scans needed to screen individuals for a hypothetical clinical trial which needs 100 abnormal amyloid individuals. For this analysis, we used the sensitivity and specificity levels that corresponded with the highest Youden cut-off. Analyses were stratified for SCD, MCI, and AD dementia diagnosis as the prevalence of abnormal amyloid individuals differs per diagnosis [21]. We assumed an average cost of 5000 USD [22] per amyloid PET scan and estimated 100 USD per plasma MDS-OAβ sample. A *p*-value < 0.05 was considered statistically significant.

**Table 1:** Subject characteristics

	Total cohort		Sample storage period (≤ 4 years)		Sample storage period (> 4 years)	
	PET-based amyloid status (n = 399)		PET-based amyloid status (n = 207)		PET-based amyloid status (n = 192)	
	Normal (n = 193)	Abnormal (n = 206)	Normal (n = 139)	Abnormal (n = 68)	Normal (n = 54)	Abnormal (n = 138)
Age, year	64.4 (6.5)	63.2 (6.6)	64.6 (6.2)	63.2 (6.5)	64.0 (7.3)	63.2 (6.7)
Female sex, n %	75 (39)	101 (49)*	54 (39)	34 (50)	21 (39)	67 (49)
MMSE (n = 393)	26 (4)	22 (5)***	27 (3.9)	22 (4.7)***	25 (3.7)	22 (4.6)***
Education, year (n = 334)	10.7 (2.9)	11.4 (2.9)*	10.5 (2.9)	11.0 (2.6)	10.9 (3.0)	11.7 (3.0)
Diagnosis						
CN^b^/MCI/AD/	70/15/11/	4/27/153/***	63/4/6/	2/10/44/***	7/11/5/	2/17/109/***
Non-AD/Other	49/48	9/13	30/36	3/9	19/12	6/4
APOE ε4 carrier (n = 389)	59 (30%)	140 (70%)***	42 (31%)	46 (69%)***	17 (32%)	94 (72%)***
CSF aβ42 (pg/ml)						
Innotest (n = 268)	1033 (246)	629 (131)***	1036 (284)	610 (182)***	1027 (184)	638 (102)***
Euroimmun (n = 60)	999 (309)	NA	999 (309)	NA	999 (309)	NA
Plasma MDS-OAβ assay^a^	0.80 (0.33)	0.97 (0.35)***	0.76 (0.32)	1.03 (0.28)***	0.89 (0.35)	0.95 (0.37)

* *p*<0.05, ** *p*<0.01, *** *p*<0.001

^a^Hemolytic samples excluded

^b^Controls include normal controls and SCD subjects

Non-AD dementia patients include possible AD, FTD, DLB, VaD and CBD, and PSP. Other includes psychiatry, other neurological diseases, postponed diagnosis or PPA. Data are presented as mean (SD) unless otherwise specified. Independent t-test or chi-squared test was performed where appropriate

Abbreviations: *NA* not available, *MMSE* Mini Mental State Examination, *MCI* mild cognitive impairment, *FTD* frontotemporal dementia, *DLB* dementia with Lewy Bodies, *VaD* vascular dementia, *PPA* primary progressive aphasia, *AD* Alzheimer’s disease dementia, *non-AD* non-Alzheimer’s disease dementia, *aβ42* amyloid-β1-42, *MDS-OAβ* Aβ oligomerization tendency, *PET* positron emission tomography

## Results

### Patient characteristics

**Fig. 1:**
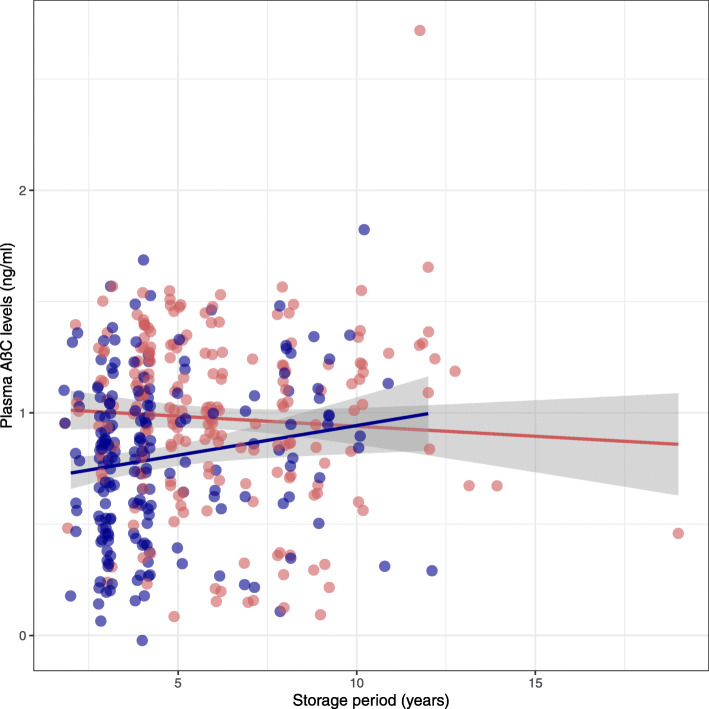
Scatterplot presents the correlation between plasma MDS-OAβ and storage period in years. Blue dots represent normal amyloid PET individuals, and red dots represent abnormal amyloid PET individuals

The average age of the total population was 63.8 ± 6.6 years old, 44% was female, and the average MMSE was 24 ± 5. Abnormal amyloid status was found in 206 (52%) subjects which included more often AD dementia subjects, whereas normal amyloid subjects included more often controls. Individuals with abnormal amyloid had lower MMSE scores and were more often APOEe4 carrier compared to individuals with normal amyloid status (Table 1). An interaction with storage period was found (*p* < 0.01; Fig. 1, Table 2), after which the cohort was stratified based on the median storage period (4 years). The groups with storage period ≤ 4 years and > 4 years did not differ from each other in patient characteristics. Both had more abnormal amyloid subjects who had lower MMSE scores and were more often APOEe4 carrier than amyloid normal subjects (Table 1).

**Table 2:** Full model for storage period and amyloid PET status for plasma MDS-OAβ

	β (se)^b^	*p*-value
Storage period, yrs	.03 (.01)	.02
Amyloid PET status^a^	.38 (.08)	< .001
Amyloid PET status × storage period, yrs	− .04 (.01)	.003

We used general linear models (GLM) with factors storage period and PET amyloid status, and their 2-way interactions

^a^Reference is normal amyloid PET status. p < 0.05 is considered significant

^b^Plasma MDS-OAβ was normalized using two-step transformation

*MDS-OAβ* Aβ oligomeric tendency, *yrs* years, *PET* positron emission tomography

### Plasma MDS-OAβ performance in samples with a storage duration < 4 years

**Fig. 2:**
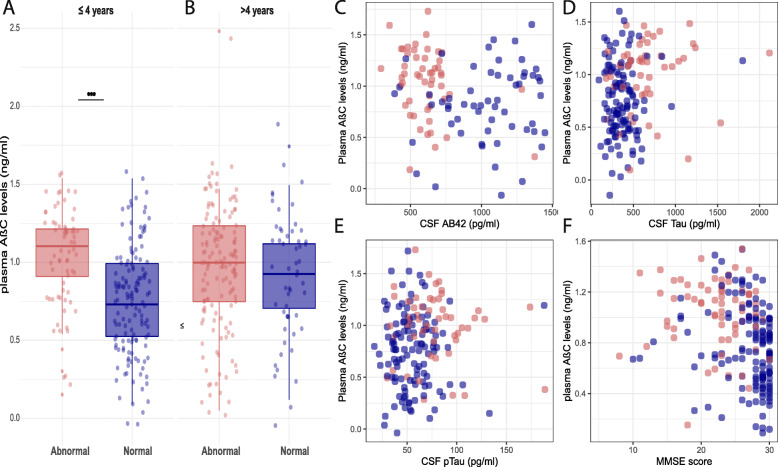
Boxplots present higher levels of plasma MDS-OAβ in abnormal amyloid PET individuals in samples with ≤ 4 years storage (n = 207) (**A**), and no change in samples > 4 years storage (n = 192) (**B**). Scatterplots present the correlation in samples ≤ 4 years storage (n = 207) between plasma MDS-OAβ and CSF biomarker concentrations: CSF Aβ 42 (**C**), CSF Tau (**D**), CSF pTau (**E**), and the correlation between plasma MDS-OAβ and MMSE score (**F**). Blue dots represent normal amyloid PET individuals, and red dots represent abnormal amyloid PET individuals. ***p-value < 0.001

**Fig. 3:**
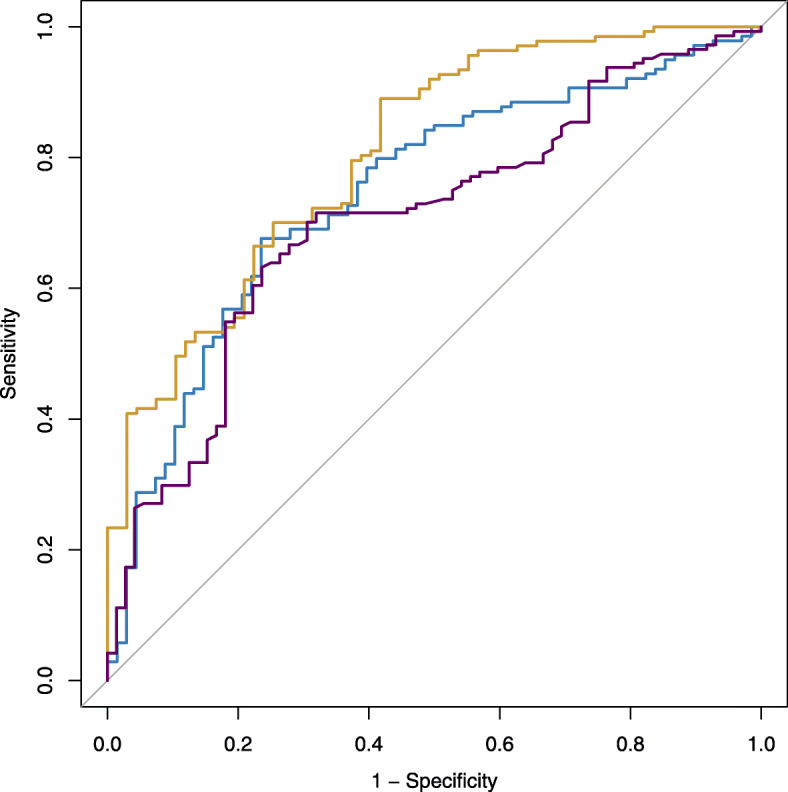
Receiver operating characteristic (ROC) curves discriminate abnormal amyloid from normal amyloid subjects as defined by amyloid PET scan based on plasma Aβ oligomer levels (blue line), age and APOEe4 genotype (purple line), and a multivariate model including APOEe4 genotype, age, and plasma Aβ oligomer levels (yellow line)

For samples with a storage period ≤ 4 years (n = 207), plasma MDS-OAβ levels corrected for age, sex, APOEe4 status, and cohort were higher in abnormal amyloid subjects compared to normal amyloid subjects (β ± se, 0.17 ± 0.05; *p* = 0.001; Fig. 1). Plasma MDS-OAβ was negatively correlated with CSF Aβ42 levels (r = − 0.20, *p* = 0.035) and MMSE scores (r = − 0.29, *p* < 0.01) and positively correlated with CSF Tau (r = 0.20, *p* = 0.01) (Fig. 2). There was no correlation with CSF pTau levels (r = 0.12, *p*>0.05). ROC analyses (Fig. 3) revealed that plasma MDS-OAβ could accurately identify individuals with abnormal amyloid PET (AUC = 0.74, 95% CI = 0.67–0.81), with a sensitivity of 76% and specificity of 67%. When combined with age and APOEe4 status the AUC increased to 0.81 (95% CI = 0.74–0.87), with a sensitivity and specificity of 58% and 89%, which performed better than age and APOEe4 genotype alone (AUC = 0.70, 95% CI = 0.63–0.78, *p* = 0.01).

### Plasma MDS-OAβ performance in samples with a long-term storage duration

We repeated our analyses in samples (n = 192) that had been stored for a longer period (> 4 years) and observed no difference in plasma MDS-OAβ levels between abnormal and normal amyloid individuals (β ± se, 0.04 ± 0.06, *p* > 0.05, Fig. 2) nor could it discriminate between abnormal and normal amyloid status (AUC, 0.50, *p* > 0.05).

### Plasma MDS-OAβ as an early predictor of amyloid status and syndrome diagnosis

Next, analyses were repeated in a pre-dementia subgroup including CN and MCI subjects (storage period ≤ 4 years; n = 78). Plasma MDS-OAβ could identify individuals with abnormal amyloid PET with an AUC of 0.77 (95% CI = 0.60–0.93), and a sensitivity and specificity of 67% and 83%, respectively. When combined with age and APOEe4 status the AUC increased to 0.86 (95% CI = 0.75–0.96), with a sensitivity and specificity of 75% and 83%, which performed better than age and APOEe4 genotype on a trend level (AUC, 0.76; 95% CI, 0.62–0.89, *p* = 0.10).

We next performed an exploratory analysis of the prediction of amyloid positivity in the non-AD and other diagnosis subgroups. The data showed largely similar results. For the comparison of abnormal amyloid PET vs normal amyloid PET, we observed an AUC of 0.72 in non-AD subgroup and an AUC of 0.75 in the other diagnosis subgroup, controlled for age and APOEe4 status.

### Exploratory cost-evaluation for plasma MDS-OAβ as a pre-screener

Lastly, we explored how the use of plasma oligomers as a pre-screen could reduce costs to find 100 individuals with amyloid pathology on a PET scan in the total group of individuals with normal cognition, MCI, or AD dementia. Given an expected prevalence of amyloid pathology of 30% in CN, 50% in MCI, and 70% in AD dementia subjects [21], the number of amyloid PET scans to find 100 amyloid positives within each group without pre-screening would be 333 (CN), 200 (MCI), and 143 (dementia). Assuming the sensitivity of 76% and specificity of 67% of the plasma MDS-OAβ test (highest Youden cut-off, 0.45) in this total cohort, MDS-OAβ needed to be determined in 439 CN, 263 MCI, and 188 AD dementia subjects to identify 195 CN, 141 MCI, and 118 AD dementia subjects with an abnormal MDS-OAβ test in order to find 100 abnormal amyloid PET cases within each clinical group. The plasma MDS-OAβ assay as a pre-screener for amyloid PET analysis would thus reduce the number of PET scans with 138 (40%) in CN, 59 (30%) in MCI, and 25 (18%) in AD dementia subjects. Considering the costs for a PET analyses and MDS-OAβ only, this would result in a cost reduction of 40% in CN, 30% in MCI, and 15% in AD dementia based on these figures.

## Discussion

We showed that plasma MDS-OAβ has good accuracy to pre-screen for brain amyloidosis in a memory clinic population, particularly when combined with APOEe4 and age (AUC > 0.80). In addition, plasma MDS-OAβ showed a negative correlation with CSF Aβ42 and MMSE, and a positive correlation with CSF Tau. Using plasma MDS-OAβ as a pre-screener resulted in reduced number of PET scans and lowered costs for amyloid screening up to 40%, which is highly beneficial for clinical trials. Of note, these results are only valid for relatively fresh samples, as a negative effect of long-term storage was found for plasma MDS-OAβ concentrations.

To date, few studies have measured Aβ oligomers levels in blood plasma, as detecting crude oligomeric Aβ in plasma is challenging owing to its low concentration in blood. Using the MDS platform, we successfully measured increased plasma MDS-OAβ levels in abnormal amyloid PET individuals compared to individuals with normal amyloid PET levels. This finding is in line with previous studies reporting increased levels of Aβ oligomers in brain tissue, CSF, and plasma of AD patients [5, 23–25]. This increase in Aβ oligomer levels is in contrast to monomeric Aβ levels, which show an evident decrease rather than increase in blood plasma [26–30]. This upregulation of Aβ oligomers could be explained by oligomerization of Aβ monomers, resulting in higher plasma Aβ oligomer levels and decreased monomeric Aβ levels. Our results also showed a correlation between plasma MDS-OAβ and CSF Aβ42, Tau, or MMSE scores, which is in line with previous plasma Aβ monomer studies [7, 26, 27, 30]. However, these correlations were not strong, and an explanation for this could be the peripheral production of plasma Aβ, by platelets, skeletal muscle cells, and other cell types [31] that contribute to circulating Aβ levels resulting in a dilution of the relation with CNS processes.

This is the first study to report on plasma AβOs as a marker for brain amyloidosis in a large amyloid PET-confirmed cohort. As the definition of AD is shifting from a syndrome to a biological construct, it is relevant to evaluate the performance of biomarkers in discriminating amyloid status [8]. One small-scale study did evaluate oligomeric assemblies of misfolded Aβ protein as a plasma marker for amyloid status between prodromal PET-positive individuals (n = 36) and healthy elderly PET-negative individuals (n = 37) [6]. Using an immune-infrared sensor method, they achieved an AUC of 0.78 (95% CI 0.68–0.88) [6]. We showed a similar good accuracy of plasma MDS-OAβ to screen for amyloid status (AUC, 0.81) in a large amyloid PET-confirmed cohort when combined with APOEe4 and age. When restricting the analyses to individuals in pre-dementia stages (i.e., CN and MCI), the accuracy of plasma MDS-OAβ combined with APOEe4 and age increased further to 0.86.

One of the suggested applications of a plasma Aβ biomarker is a screening test for brain amyloidosis in specialized memory clinics or for clinical trial inclusion [32]. Previous studies have been successful in identifying amyloid status with high accuracies (AUC, 0.79–0.97) using various types of plasma Aβ markers [26–30, 33] and plasma pTau isoforms [34–38]. The plasma MDS-OAβ assay had similar or somewhat lower accuracies compared to these other plasma biomarker tests. While techniques used in some studies, such as immunoprecipitation mass spectrometry [28, 29], are labor-intensive and time-consuming, the Simoa assays and the MDS method allow high-throughput analysis. The MDS method highly resembles an ELISA in simplicity and automation possibilities [7, 18, 39] and as such, allows broad implementation. Another added value of our study is that we have tested the plasma MDS-OAβ assay in a heterogeneous cohort including other neurodegenerative or neuropsychiatric disorders besides the clinical AD spectrum, while previous plasma Aβ included primarily cohorts which contained the clinical AD spectrum (i.e., healthy controls, MCI, or AD dementia). The heterogeneity of the cohort used in this current study better resembles a memory clinic population, the setting where plasma biomarkers will likely be applied in the future to pre-screen for brain amyloidosis. Additionally, using plasma MDS-OAβ as a pre-screener in a hypothetical clinical trial scenario lowered the number of PET scans up to 40% depending on clinical diagnosis. Therefore, plasma MDS-OAβ could be beneficial for pre-screening in clinical trial settings, as it could potentially reduce costs.

It is well known that pre-analytical factors concerning sample handling and processing can influence the measured concentration of (plasma) biomarkers, therefore leading to variability in results, preventing establishment of a universal cutoffs and between-laboratory comparisons [40, 41]. We previously observed a negative effect of long-term storage on plasma MDS-OAβ levels upon visual inspection (data not published) and, therefore, decided to evaluate this in the current study. We found that for samples with a storage time > 4 years plasma MDS-OAβ levels no longer differed between normal and abnormal amyloid individuals. Our finding is not fully in line with one recent study that investigated the long-term storage effect on plasma monomeric Aβ and found stable plasma Aβ levels after long-term storage up to 5 years at – 80 °C [42]. This discrepancy might be caused by the difference in storage length between the previous study (up to 5 years) and the current study (up to 19 years). It might also be caused by the difference in analytical methods (MDS vs. IMR) or the different Aβ species (MDS-OAβ vs (in principle) monomeric Aβ42). It could be the case that plasma MDS-OAβ levels of normal amyloid increase over time and reach similar levels as plasma MDS-OAβ levels of abnormal amyloid individuals, whereas in monomeric Aβ42 this does not happen. It could be hypothesized that long-term storage might induce stress on the oligomeric Aβ42 protein which results in perturbation of the protein and an increased aggregation tendency in normal amyloid individuals, which does not occur in abnormal amyloid individuals as they have already reached maximum oligomerization. A similar increase in protein aggregation induced by protein-stress has previously been reported after freeze-thawing [43]. The effect of long-term storage time implies that the plasma MDS-OAβ assay cannot be used to perform research projects with samples that have been stored in biobanks for a long period. This novel finding could be of interest to other research groups interested in measuring plasma biomarkers of amyloid. Additional pre-analytical testing is needed to determine the precise maximum storage period and to compare the effect of long-term storage with other types of blood-based Aβ biomarkers. Nonetheless, as in daily clinical routine, fresh blood samples are used; we do not expect this will present a problem for daily clinical practice. This is supported by recent results from a systematic study into pre-analytical stability, showing no effect of up to 2 weeks storage at either room temperature or – 20 °C on the plasma MDS-OAβ levels (manuscript under review).

Our study has several strengths including our large well-defined amyloid PET-confirmed memory clinic population. In addition, CSF and plasma collection follows a highly standardized protocol in our center, thus minimizing confounding effects in pre-analytical processing. Moreover, the oligomerization assay technique developed for this plasma MDS-OAβ assay can potentially be employed for other proteinopathies as well, such as α-synuclein which is often seen in dementia with Lewy bodies. This might result in a screening panel of plasma biomarkers for different types of neurodegenerative disease. Among the limitations of our study is that the plasma MDS-OAβ assay is not yet available on an automatic platform, thus enhancing the risk for analytical variation. However, automation is currently under development, further facilitating broad implementation and minimizing analytical variation. In addition, plasma MDS assays for other AD biomarkers, such as phosphorylated Tau, are currently under development to further capture the full pathological profile of AD [8]. Lastly, it would be interesting to study the association between plasma MDS-OAβ with specific cognitive domains, through elaborate neuropsychological testing, to get an in-depth understanding of the association between plasma MDS-OAβ and cognitive impairment.

## Conclusions

In conclusion, plasma MDS-OAβ has the potential to be used as a pre-screener for brain amyloidosis in large heterogeneous memory clinic populations. The advantages of the low-cost MDS-OAβ blood test include the ease of blood collection over a lumbar puncture or a costly PET scan. Additionally, using plasma MDS-OAβ as a pre-screener based on the results of this current study reduced the number of amyloid PET scans needed and lowered total costs up to 40%, highlighting a potential use for clinical trials settings. In addition, the novel finding of long-term storage duration on plasma MDS-OAβ levels could be of interest to other research groups interested in measuring plasma biomarkers of amyloid.

## Data Availability

Data are available from the authors upon reasonable request.

## Abbreviations

AD: Alzheimer’s disease
ADC: Amsterdam Dementia Cohort
Amsterdam UMC: Amsterdam University Medical Centers
APOEe4: Apolipoprotein epsilon 4
AUC: Area under the curve
Aβ: Amyloid beta
Aβ42: Amyloid Beta 1-42
CSF: Cerebrospinal fluid
CV: Coefficient of variation
EDTA: Ethylenediamine tetraacetic acid
EEG: Electroencephalography
ELISA: Enzyme-linked immunosorbent assay
EMIF-AD: The European Information Framework for AD
GLM: General linear models
MCI: Mild cognitive impairment
MDS: Multimer detection system
MDS-OAβ: Amyloid oligomerization tendency measured by the multimer detection platform
MMSE: Mini Mental State Examination
MRI: Magnetic resonance imaging
NC: Normal controls
OAβ: Amyloid oligomerization tendency
PET: Positron emission tomography
pTau: Tau phosphorylated at threonine 181
SCD: Subjective cognitive decline
Tau: Total Tau
USD: US Dollar
